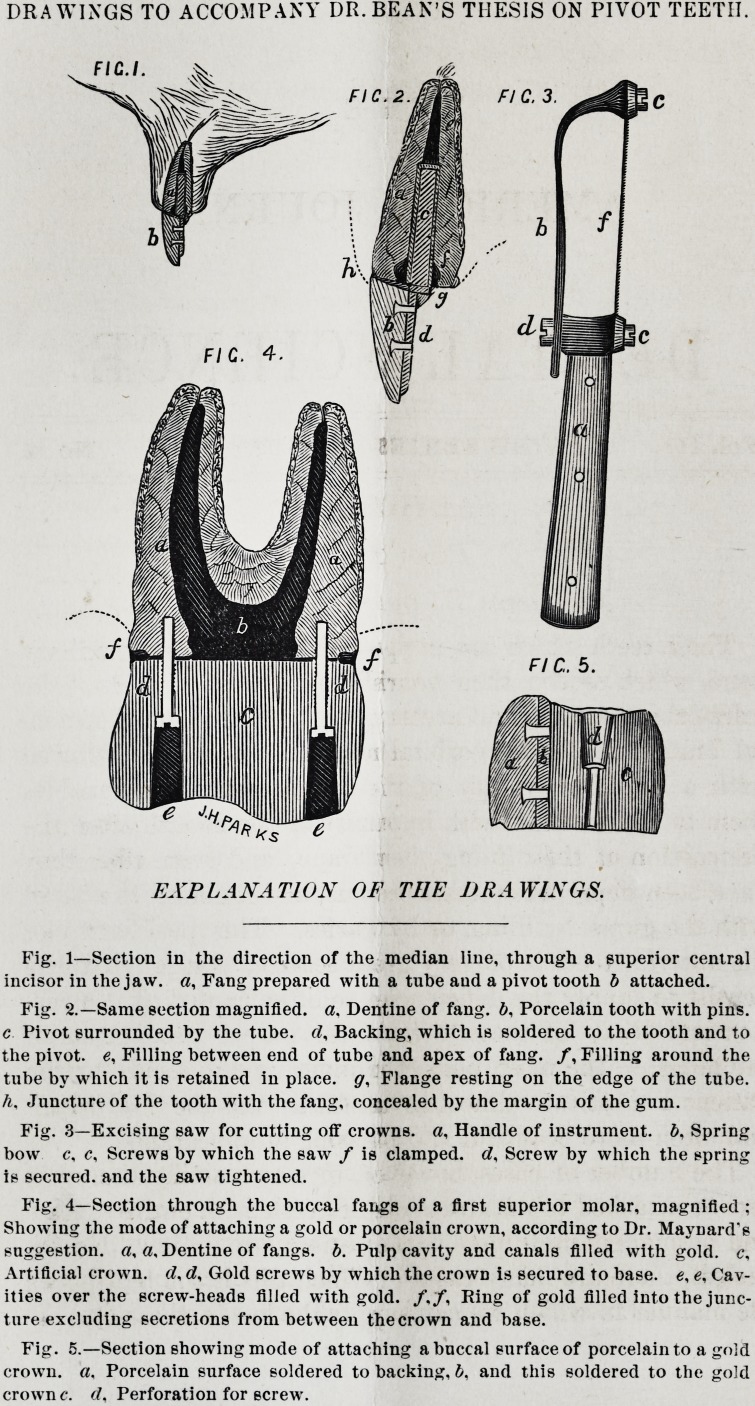# Pivot Teeth

**Published:** 1869-08

**Authors:** James B. Bean


DRAWINGS TO ACCOMPANY DR. BEAN'S THESIS ON PIVOT TEETH.

FIG. 1.

FIG. 2.

FIG. 3.

FIG. 4.

FIG. 5.

### *EXPLANATION OF THE DRAWINGS.*

---

Fig. 1—Section in the direction of the median line, through a superior central incisor in the jaw. *a*, Fang prepared with a tube and a pivot tooth *b* attached.

Fig. 2.—Same section magnified. *a*, Dentine of fang. *b*, Porcelain tooth with pins. *c* Pivot surrounded by the tube. *d*, Backing, which is soldered to the tooth and to the pivot. *e*, Filling between end of tube and apex of fang. *f*, Filling around the tube by which it is retained in place. *g*, Flange resting on the edge of the tube. *h*, Juncture of the tooth with the fang, concealed by the margin of the gum.

Fig. 3—Excising saw for cutting off crowns. *a*, Handle of instrument. *b*, Spring bow *c*, *c*, Screws by which the saw *f* is clamped. *d*, Screw by which the spring is secured. and the saw tightened.

Fig. 4—Section through the buccal fangs of a first superior molar, magnified; Showing the mode of attaching a gold or porcelain crown, according to Dr. Maynard's suggestion. *a*, *a*, Dentine of fangs. *b*. Pulp cavity and canals filled with gold. *c*, Artificial crown. *d*, *d*, Gold screws by which the crown is secured to base. *e*, *e*, Cavities over the screw-heads filled with gold. *f*, *f*, Ring of gold filled into the juncture excluding secretions from between the crown and base.

Fig. 5.—Section showing mode of attaching a buccal surface of porcelain to a gold crown. *a*, Porcelain surface soldered to backing, *b*, and this soldered to the gold crown *c*. *d*, Perforation for screw.

THE

### AMERICAN JOURNAL

OF

### DENTAL SCIENCE.

Vol. III. THIRD SERIES--AUGUST, 1869. No. 4.

### ARTICLE I.

### *Pivot Teeth.*

By James B. Bean, D.D.S.,M.D.

Those teeth which are supported by the inter-maxillary bone, which receive their nourishment from a branch of the infra-orbital artery, and are supplied from the anterior dental branch of the infra-orbital nerve, seem to be endowed with a peculiar vitality of their periosteum that enables them to be retained with impunity in the mouth after the destruction of their lining membrane, and even after they have been deprived of their crowns, and reduced to a level with the gums by filing or by caries. This peculiarity of the anterior teeth is of very great service, as it enables the dentist to supply the deficiencies so often caused by destruction and decay of these teeth, after proper preparation of the fangs, in the most perfect manner, and to secure to the patient a substitute almost as useful as was the lost organ, and often more beautiful and pleasing in appearance.

The number of cases, however, in which artificial crowns can be attached to natural roots of teeth with entire success and permanent usefulness to the patient, are limited. Nevertheless, there are many cases in which this is the best possible manner in which we can supply the loss of these organs.

The utmost resources of our art, therefore, should be brought to bear in producing a substitute that will give such entire satisfaction as a properly constructed *pivot tooth.*

In the cases for which we are called to operate, the crowns of the teeth under consideration have either been destroyed or disfigured by mechanical violence, or have been corroded by caries to such an extent that restoration by filling with gold would be impossible, or at least inadmissible. In the first class of cases, if the accident has not produced contusion of the periosteum, and consequent loosening of the tooth, and the fang has not been fractured below the margin of the gum, the operation may be performed at once, with the most complete success. And sometimes, after considerable inflammation of the periosteum, and consequent loosening (where the concussion has been severe), the tooth will in a short time become sufficiently firm in its socket to warrant the success of the operation. The second class is of more frequent occurrence, and if the teeth yet retain their vitality, and are firmly fixed in their socket, surrounded by a healthy gum and periosteum, the chances of success are most favor able, and, if proper pains be taken, the best results will follow.

The peculiar advantages claimed for pivoted teeth, are, that they can be immediately applied without the usual delay dependent on the absorption of the alveolus, and without encumbering the mouth with plates, clasps, &c., while at the same time they are much firmer, and more useful in the mouth than plate teeth.

The facility with which this operation may be performed has caused it to be resorted to by many unskillful practitioners, under the most unfavorable circumstances, by reason of which, it has gone into disrepute with many intelligent dentists. Yet the best operators, and the most eminent dentists who have ever graced the profession have given it their sanction, and says Prof. Harris, "It is certainly the best method that can be adopted for replacing the loss of the six upper front teeth."

Many different methods have been proposed for the attachment of artificial crowns to roots of natural teeth, but before attempting any of them, certain preparations and precautions are necessary. The favorable indications already enumerated should be present if possible, and all diseased action in other parts of the mouth should be corrected by the removal of all old fangs and such diseased teeth as cannot be restored to a healthy condition, and the gums and living membrane of the mouth should be freed from all abnormal conditions by the removal of exciting causes, and the use of the proper remedial agents indicated. Moreover, the patient should have a sound constitution, free from any cachectic habit that might be prejudicial to success

The method of fastening crowns of natural teeth to fangs in the mouth by means of pivots or tenons of wood, was practiced in the remotest antiquity, and has been described by the oldest dentists who have written on the subject of artificial teeth; and Bourdet, who wrote in Paris more than a hundred years ago, describes the manner of attaching a crown by wooden, and also by metalic pivots or tenons.

Since the introduction of porcelain teeth, this method has been practiced to a very great extent, and oftentimes by the hands of incompetent and inexperienced operators. The common practice is to merely mark the tooth with a file at the margin of the gum, clipp off the crown with the excising forceps, extirpate the nerve with a drill and file to a level with the gum. And after filing the porcelain crown, and enlarging the canal of the root, a hickory pivot forced into each, driving the tooth close up to the gum, completes the operation, and the patient goes off highly pleased. But this pleasure is generally of short duration, for in a large number of cases so treated, inflammation of the periosteum, and suppuration of the remaining portions of the pulp ensues, and troublesome alveolar abscess is the result; often remaining as long as the tooth is in place. Numerous contrivances have been proposed to obviate this very serious difficulty; such as grooves and perforations in the pivot for the escape of the purulent secretions. But there are so many serious objections to the use of wooden pivots in any of these forms, that they have been almost entirely laid aside by the bests operators in the profession.

There are several different modes of attachment by metalic pivots that have been practiced by eminent operators, which are superior in most cases to wood alone. The English dentists have practiced for a long time the mode of attaching the tooth to a gold wire, wraping this with silk, and after being saturated with a thick solution of mastich, it is forced into the canal of the previously prepared fang. Others have used a simple gold pivot fitted into the root; but these soon become loose by wearing, and the action of the fluids of the mouth on the walls of the canal, and to obviate this, some have filled the canal securely with a pin of hard wood, and then drilled into the centre of this for the introduction of a close-fitting gold pivot. This method affords a very firm hold, and a tooth fitted in this manner will last many years, under favorable circumstances.

In all the foregoing methods described, the walls of the canal in the root to which the tooth is attached, are necessarily exposed to the action of the fluids of the mouth, and these by decomposition, become corrosive agents, gradually enlarging the canal, and finally entirely unfitting it for the retention of an artificial tooth. To prevent this destruction of the fangs, a method has been adopted of securing a gold tube in the canal, into which the gold pivot is accurately fitted, thereby making a very perfect operation. The manner of effecting this, will now be described in detail, and also the manner of attaching artificial crowns to molar teeth.

If the tooth still retains its vitality, the pulp being exposed, and the patient will not submit to its extirpation with an instrument, we at once decide on destroying its vitality by the application of arsenious acid, and this can better be done before the removal of the remaining portions of the crown.

The arsenious acid should be combined with twice its weight of sulphate of morphia, and intimately mixed with a sufficient quantity of creosote to form a thick paste, and having saturated a very small pellet of cotton with the composition, it is applied directly to the exposed pulp, and the remainder of the cavity filled with wax and cotton, as directed by Dr. Maynard, or with tin foil packed moderately close. This is permitted to remain from ten to twenty-four hours, when the nerve will generally be found to have lost its vitality, and can be removed without pain, sometimes coming away entire.

If the tooth has already lost the vitality of its lining membrane, or as soon as its destruction is affected by the arsenic, or if the patient will submit to its extirpation by mechanical means, we proceed at once to remove the remaining portions of the crown ; and this should be done with the greatest care, so as to prevent injury to the alveolo-dental periosteum, thereby lessening the probabilities of success. The best method is to use an instrument constructed somewhat like a file-carrier, with a very narrow main-spring saw about two inches in length, properly secured with clamps and screws, to a small bow similar to the saw-frame used by jewellers, except that the saw should cut at right angles to what it does in that instrument. With this contrivance the entire crown, or any portion of it can be removed without danger of fracturing the fang or injuring the investing membranes. Where the nerve still retains its vitality, the first contact of the saw would be productive of intense pain : therefore we should saw half through the dentine on all sides of the tooth, then clip off the crown with the excising forceps.

Having sawed off to as near as possible the proper length, the next operation is to remove any remaining portions of the nerves and blood vessels, and file off the end of the stump to a smooth surface, to within about one half, or one fourth of a line from the margin of the gum. Round off the sharp edges of enamel on the posterior and approximal portions, and bevel the anterior edge a little below the margin of the gum, giving it a slight concave appearance, so as to accommodate the neck of the plate tooth which is to rest against it. It is better at this stage of the operation, to stop the canal loosely with a pellet of cotton or floss silk saturated with spirit of camphor, and dismiss the patient for two or three days, when, if no inflammation be present, the canal may be cleaned out, and carefully filled, from the apex to within four or five lines of the orifice with gold foil. Any favorite method of fang-filling may be employed, but one of the best is to carefully introduce the first piece of gold in the form of a conical shaped "cylinder," rolled on a small five-sided broach till it is of a size and shape to fit the canal, then force it well to the apex with a straight fang-filling instrument; the remainder is then filled with pellets or ropes as usual. The remaining portion of the canal not filled, should now be enlarged to about one line in diameter, if the size of the fang will admit of it, down to the gold filling, making the bottom smooth and solid, and the sides parallel. The orifice, to the depth of nearly a line, is again enlarged with a bur to about two lines in diameter, and a small groove, or undercut is formed around the margin for the retention of the gold filling subsequently to be introduced around the tube.

For the tube, either the hollow gold wire described by Prof. Harris in his "Principles and Practice," or simple gold tubes, made of gold plate may be employed. If the latter be chosen, they may be formed by bending a piece of ordinary gold plate around a wire, so as to form a cylinder sufficiently large to fit the smaller portion of the canal prepared for it. Then solder with gold solder as fine as can be used. A piece of the tube half an inch in length should now be cemented with shellac into a hole bored through a piece of wood half an inch in thickness to serve for a handle, while the interior is carefully dressed out with a jeweller's broach which has a slight taper, making it smooth and regular within. A solid gold wire pivot is now carefully filed and fitted by grinding with fine emory and water, making a "ground joint," whereby the pivot is firmly held when in place. Any portion of the wire that may now project beyond the smaller end of the tube, should be cut off even, while at the larger end it should project at least one fourth of an inch.

The tube must now be taken out of the cement, and a piece of plate soldered to the smaller end, forming a *bottom.* An easier flowing solder should be used for this, so as not to disturb the first. This tube, or *dress cap,* thus formed, after being "pickled," and filed off smooth, is ready to be inserted into the fang.

Some have proposed to cut a screw on the tube with a screw-plate, and with a tap to cut a corresponding thread in the fang, whereby the tube is firmly secured in its place, and filled around with gold. But the most convenient way is to cut a number of barbs with a sharp knife on the outside, looking toward the open end, so as to securely retain it in place when filled around with gold. Being made so as to go into the root rather loosely, several folds of gold foil are wrapped around it, and after carefully drying the parts with bibulous paper, the pivot being in its place in the tube, the whole is forced to the bottom of the cavity, and the loose porticns of foil cleaned away from around it.

Having previously prepared some adhesive foil, and suitable instruments all ready, keep the tube and root free from moisture until the space around the former is perfectly filled with gold, and perfectly consolidated. The gold pivot is now removed, and the tube carefully sawed off nearly level with the end of the fang. Then by filing and consolidating the whole must be made smooth, and the surface and edges of the end of the fang well polished. We now have the fang perfectly preserved, with a good filling, and a gold tube firmly secured in it, with a gold pivot accurately fitting the latter.

The next operation then is to secure a suitable tooth to the pivot, and for this purpose a plain plate-tooth is selected that will fulfil the requirements of the case in size and shape; the color, of course, should be as near the same shade of the adjoining teeth as possible—even a shade darker would form a less conspicuous contrast than any lighter. This tooth should be ground and fitted to the beveled edge previously formed on the anterior side of the root, so as to have the free margin of the gum cover the point of union. Then after soldering and finishing up a strong backing upon the tooth, it is fitted into its proper position, with the gold pivot in place, on which has been soldered a small shoulder or ring of plate, and the projecting portion of the wire cut off. The best method of securing the shoulder in its proper position, is to cut out a disk of gold plate larger than the diameter of the pivot, which is perforated with a hole just large enough to admit the pivot up to the point at which it is to be soldered—and this should be a little less than the depth of the tube—and being retained at this point, it is made to fit closely down on the fang, the whole then carefully withdrawn and placed up to the ring in plaster and asbestos. If the ring be loose on the pivot it must be kept in place by a bit of wax or plaster while withdrawing them from the tube, and as soon as the investment of plaster and asbestos is dry the wax is removed, borax applied, and the piece soldered with *fine* solder. The pivot is now tried again in the mouth and if the fit be satisfactory, the projecting portion is cut off and any prominence filed smooth so the backing and the tooth can be properly adjusted to it The tooth can now be attached to the pivot by a small portion of shellac, again tried in, and altered if necessary till the position is satisfactory. If the pivot does not fit too tightly, the whole can be withdrawn together, entirely invested in plaster and asbestos, except the portion on which the solder is to flow, and when dry, the cement is carefully removed, borax applied and the union strongly soldered.

The piece is now finished up, reducing the shoulder around the pivot to less than half a line in breadth, as a large plate covering the end of the fang would be of no advantage, but would only form a lodgement for foreign matter and the secretions of the mouth, whereby decomposition and consequent destruction of the dentine of the fang would be the result. If the pivot is not retained sufficiently firm in the tube, it may be wrapped with a very few fibres of floss silk or cotton, and when forced into its place with a slight rotary motion, it will remain quite firm, and can be used by the patient with almost as much satisfaction as a natural tooth. If the adjustments have been properly made, the shoulder or flange will fit up closely and rest on the edge of the tube, the neck of the tooth resting on the beveled edge made for its accomodation, thereby preventing the tooth from turning on its axis, and the juncture is hidden by the free margin of the gum.

Artificial teeth attached by this method, if the operation be faithfully performed and the patient take proper care of it afterwards, may be retained and used with the greatest satisfaction, for many years. They should be removed at least three times a week, the parts thoroughly cleaned, and the pivot if necessary, wound with new fibres of cotton or floss silk before returning it to its place. Proper care and cleanliness will entirely prevent any recurrence of decay in the fang, and the appliance is far more permanent and less inconvenient than any other kind of dental substitute.

The principle as above described, can only be used in attaching artificial crowns to the six anterior upper teeth, and perhaps the lower cuspids; the fangs of the lower incisors being too much compressed to permit the introduction of a tube of sufficient size. The molars and bicuspids, however, may be accomodated by a modification of the process, using two or three smaller tubes instead of one, with corresponding pivots soldered to the gold base to which the teeth are attached. But the better way of treating these teeth, especially the molars, where an artificial crown is called for, is to adopt a plan suggested by Dr. Maynard of Washington City. This consists in making a crown of solid gold, or of porcelain, and attaching it to the previously prepared base by means of screws, and then filling around the juncture a ring with adhesive gold, or common gold foil, thereby excluding the secretions of the mouth, and making a much easier and more perfect operation than by building on an entire crown with adhesive gold.

The root or base should present all the healthy indications before enumerated in the case of incisors, and each fang should be thoroughly and carefully filled, together with the pulp cavity, or as much of it as is left, and the whole base is then made smooth and polished. Then with a proper instrument a small shallow groove is cut around the margin of the base to retain the filling; it is then ready for the attachment of the crown.

A model of wax should be made, fitting the basement already prepared, properly articulated with the tooth above, and of the desired shape of the crown to be attached. From this model is made a mould or matrix of fine sand and plaster, and, after thoroughly drying, the gold may be poured in in a melted state from the crucible, the mould having been previously warmed. This may now be finished up and polished, and two or three holes drilled vertically through it so as to come on to the most solid portions of dentine in the base. These are deeply countersunk at the coronal aperture, to give place for the heads of the screws. A similar crown may be carved of porcelain, so as to represent the natural tooth, having the apertures for the screws drilled through before baking. Or merely a buccal surface might be made by the manufacturers, furnished with platinum pins by which it could be soldered to the gold crown, where the operation would be likely to be exposed to view in the mouth.

The crown, whether of gold or porcelain, is now placed on the base, which it should fit accurately, and the places for the screws marked and the holes drilled. Gold screws are made of the proper length, and with a tap, threads are cut in the base for the accomodation of each screw. A small groove or undercut is made with a graver around the periphery of the base of the crown, corresponding to the one already made in the base itself, for the retention of the gold filling.

All the parts are now thoroughly dried, and having all the requisite appliances for completing the operation at hand, the crown is securely screwed to the base, and the groove at the juncture carefully filled with gold foil; also filling over the heads of the screws as in common crown cavities, thereby excluding entirely, all foreign matter or buccal secretions. The surfaces of the fillings are now finished, and the whole operation completed.

These operations necessarily require a considerable amount of patience and skill in their performance; but there are many cases in which they are called for either by the necessity or preference of the patient, and whatever can be done by the dentist toward the preservation or restoration of the natural teeth, without encumbering the mouth with plates, clasps, &c., should be considered the highest productions of his professional skill. And where a very useful organ can be restored to do good service by such means, they should not be considered "fancy operations" but the utmost resources of scientific knowledge, and mechanical skill should be brought into requisition, to produce results *as near perfection as possible.*

---

## Figures and Tables

**Figure f1:**