# Erratum

**Published:** 1869-09

**Authors:** 


*Erratum.*—In Dr. Beans' article on "Pivot Teeth," published in the August No. of the *Journal*, on page 157, line 7, instead of *dress cap* read *deep cup*. Absence from home prevented our reading the proof sheets of this article, otherwise such an error would not have occurred.

